# Isolation and Pathogenic Characterization of Pigeon Paramyxovirus Type 1 via Different Inoculation Routes in Pigeons

**DOI:** 10.3389/fvets.2020.569901

**Published:** 2021-02-17

**Authors:** Han Chang, Shengyong Feng, Yutian Wang, Fuhuang Li, Qianqian Su, Bo Wang, Juan Du, Hongxuan He

**Affiliations:** ^1^National Research Center for Wildlife-Borne Diseases, Institute of Zoology, Chinese Academy of Sciences, Beijing, China; ^2^College of Life Sciences, University of Chinese Academy of Sciences, Beijing, China; ^3^Department of Microbiology, Beijing General Station of Animal Husbandry, Beijing, China

**Keywords:** pigeon, paramyxovirus, virus, Newcastle disease, pathogenicity, route

## Abstract

Pigeon paramyxovirus type I (PPMV-1) causes regular outbreaks in pigeons and even poses a pandemic threat among chickens and other birds. The birds infected with PPMV-1 mainly show a pathological damage in the respiratory system, digestive system, and nervous system. However, there were few reports on the efficiency of the virus entering the host *via* routes of different systems. In the present study, a PPMV-1 strain was obtained from a dead wild pigeon in 2016 in Beijing, China. The mean death time (MDT) and the intracerebral pathogenicity (ICPI) of our isolate showed medium virulence. Phylogenetic analysis based on F gene sequence showed that the isolate belonged to subgenotype VIb, class II, which dominated in China in recent years. Then, we evaluated the infection efficiency of different routes. Pigeons were randomly divided into five groups of six as follows: intracephalic (IC), intranasal (IN), and intraoral (IO) infection routes, cohabitation infection (CO), and negative control (N negative). All pigeons were inoculated with 100 μl·10^6^ EID_50_ PPMV-1 virus. After infection, pathological lesions, virus shedding, body weight change, survival rate, and tissue tropism were tested to compare the efficiency of the different infected routes. The mortality of groups IC, IN, IO, and CO were 100, 66.7, 50, and 33.3%, respectively. Weight loss in group IC was higher than the other groups, followed by groups IN and IO. The lesions observed in PPMV-1-infected pigeons were severe, especially in the lung and intestine in group IC. Viral shedding was observed from 2 dpi in groups IC and IN, but the shedding rate was higher in group IN than group IC. The longest period was in group CO. Tissue tropism experiment showed that our isolate has a wide range of tissue distribution, and the virus titer in the heart and intestine of group IC and in the brain of group IN was higher. Our data may help us to evaluate the risk of transmission of PPMV-1.

## Introduction

Newcastle disease, caused by Newcastle disease virus (NDV), is classified as a notifiable disease by the World Organization for Animal Health (OIE), because of its high morbidity and mortality in avian species. At least 250 avian species, including chickens, ducks, and pigeons were reported to be susceptible to NDV (1–3). NDV is a single-stranded, negative-sense, and non-segment RNA virus, which belongs to the genus Orthoavulavirus, subfamily Avulavirinae, family Paramyxoviridae of the Mononegavirales order (4–7). NDV isolates have been classified into classes I and II. NDV of class I are mostly of low virulence and contain only one genotype (8), and class II virus consists of 18 genotypes (9–13) based on the phylogenetic analysis of nucleotide sequence of the F gene and genomic size (14, 15). Pigeon paramyxovirus type 1 (PPMV-1) found in birds of Columbidae family, mainly turtle dove (*Streptopelia turtur*) and Eurasian collared dove (*Stretopelia decaocto*) is a variant of Newcastle disease virus, and it is almost genotype VI, class II. PPMV-1 first reported in England during the late 1970s, affected racing pigeons with outbreaks in domestic chickens (16), which caused the third worldwide pandemics of NDV (17). In recent years, a high mortality (from 40 to 80%, even to 100% in some cases) caused by PPMV-1 has been observed in pigeons (18–22). Clinical signs of the infected birds involve nervous, respiratory, and digestive system symptoms (23), consisting of moderate to severe depression with neck twisting, ataxia, crouch, paralysis, eyelid edema, diarrhea, and green loose feces. NDV has been documented to remain infectious in feces and carcasses for at least a couple of weeks, several months in feathers, and up to 90 days in soil or water (24). Quite a few times, outbreaks in poultry have been ascribed to PPMV-1 (25–28). The virulence can be enhanced after serial passages in chickens (17, 21, 29, 30), which makes these pigeon-originated viruses a tremendous and continuous threat to the poultry industry (31–33). For these reasons, the potential transmission of the virus and the effective route where the virus will enter the host have been considered points of concern. In the present study, the pathogenicity of a PPMV-1, obtained from a dead wild pigeon in 2016 in Beijing, China, was investigated *via* different inoculation routes. Findings from our study showed intracephalic, intranasal, and intraoral infection routes were effective, but intracephalic was the most.

## Materials and Methods

### Viral Isolation, Amplification, and Full-Length Genome Sequencing

A moribund pigeon with neck twisting, diarrhea, and leg paresis or paralysis was found in Beijing, 5 September 2016. We initially diagnosed that the pigeon was infected with PPMV or avian influenza virus based on clinical symptoms, then, the avian influenza virus was excluded and PPMV-1 infection was confirmed by RT-PCR. The identification of Newcastle disease and the separation of strains are as follows: viral RNA was extracted from the tissues of the pigeon (i.e., heart, liver, spleen, lung, kidney, stomach, brain, trachea, intestine, and pancreas) using Trizol reagent (ambition by Life Technologies, Beijing, China) according to the manufacturer's instructions. Reverse transcription was performed as described (34). The detection gene (a part of fusion gene, 486 bp) was amplified from the cDNA by PCR utilizing Taq DNA Polymerase (CWBIO 2× Taq MasterMix, Cat. CW0682M), and the primers were designed according to the conserved sequence (34) (Primer sequence: F:CAGCTGCGGCCCTAATACA; R:TGGATGCCCAAGAGTTGAG). The program was as follows: 95°C for 5 min; 35 cycles of denaturation at 95°C for 30 s, annealing at 55°C for 30 s, and extension at 72°C for 25 s; and a final extension at 72°C for 10 min. The PCR products were visualized by 1% agarose gel electrophoresis. Other pathogens (circovirus, avian influenza, and pathogenic bacteria) were negative. The viruses from different organs were plaque-purified three times on primary chicken embryo fibroblasts and inoculated into the allantoic cavity of 9-day-old specific-pathogen-free (SPF) eggs. The virus was isolated and RNA was extracted from the allantoic cavity, detected using PCR as abovementioned. The strain was designated as PPMV-1/pigeon/Beijing/China/01/2016 and abbreviated as PPMV-1/BJ-01/CH. Complete genome of our strain was amplified (primers are shown in Supplementary Table 1), sequenced, and submitted to GenBank (GenBank ID: MH807446).

### Virulence-Test

There are three virulence evaluation indexes on Newcastle disease virus, and virulence is usually determined by no <2 indexes. The mean death time (MDT) was determined in 9-day-old SPF chicken embryo eggs, and the intracerebral pathogenicity index (ICPI) was determined in 1-day-old chick as previously described (35). The least-fatal dose and egg-infectious dose of the virus were tested in 9-day-old SPF chicken embryo eggs by multiple proportion dilution (from 10^−3^ to 10^−9^) with five repeats per dilution and calculated using the Reed and Muench method.

### Phylogenetic Analysis Based on Complete F Gene

To determine the genetic relationships of the PPMV-1/BJ-01/CH isolate to others, a phylogenetic tree was constructed using the MEGA 7.0 software with the maximum likelihood method *via* the Kimura two-parameter model based on the complete F gene from 21 different subgenotypes of the reference PPMV-1 isolates and 1 APMV-1 as outgroup (8, 12, 29). All complete sequences of the F gene were downloaded from GenBank and aligned utilizing ClustalW. Nucleotide sequences obtained in the present study were aligned with reference sequences available in the GenBank database to determine the subgenotypes.

### Animal Experimental Infection

To further determine the pathogenicity of the virus, a total of 31-month-old pigeons, with approximately equal body weight of −5–5%, were used in our study. These pigeons were bought from a hatchery in Miyun District, Beijing, and certified by hemagglutination inhibition (HI) experiment to have no antibodies of NDV and AIV. These pigeons were randomly divided into five groups, and a marked group was placed in a separate cage in an animal room under biosafety conditions. Adequate food and drinking water were provided. Pigeons were inoculated with the virus by intranasal, intraoral, intracephalic, and cohabitation infections (signed as groups IN, IO, IC, and CO, placing groups CO and IC together), which represented through respiratory system, digestive system, nervous system, and natural infection, respectively. Additionally, the negative control group received phosphate-buffered saline (PBS) solution at pH 7.2. All infected groups were inoculated with a dose of 10^6^ median embryo lethal dose (ELD50)/100 μl each, calculated using the Reed and Muench method. Subsequently, all pigeons were observed daily for clinical signs, and clinical symptoms, mortality, and morbidity were recorded. Oropharyngeal and cloacal swabs were taken every day, and body weight was determined every other day until day post-infection (dpi) 14 (0 day post-infection in group CO means the day the pigeons were infected), when there is only one group left to shed virus.

### Virus Shedding

All of the oropharyngeal and cloacal swabs were collected, placed in tubes with phosphate-buffered saline solution and 2% fetal bovine serum and stored at −80°C until RNA extraction. RNA was extracted, and reverse transcription and RT-PCR were performed as above to test the virus shedding. In addition, cDNA of the isolate in this study and reagent-grade water were used as positive and negative control, respectively.

### Gross Lesion

Lesion formation and lesion size are often used to quantify virulence (36). In our study, organs of all dead pigeons were collected on dpi 5 and 14. The collected tissues (including the heart, brain, lung, intestine, and liver) were pathologically lesion examined. Individuals that died ahead of time point were examined and recorded in advance. Positive rate was tallied by percentage.

### Histopathology

Tissues from each group were collected and fixed with 10% neutral formalin. The sections were stained with hematoxylin and eosin (HE), and all HE stained sections were examined for the presence of microscopic lesions.

### Tissue Distribution

To understand the distribution and virus load of PPMV-1 in organs from different groups at dpi 5 when clinical symptoms emerged, we randomly choose three from each group, and collected samples from the heart, brain, lung, intestine, and liver. The virus was isolated from tissues of the same weight, and TCID_50_ in DF-1 cells (chicken fibroblast cell line) was used to estimate viral loads of the five groups; 3 × 10^4^ DF-1 cells were seeded in 96-well plate with five repetitions 1 day before infection. Twenty-four hours later, the cells were infected with different dilutions of the virus for 1 h at 37°C with shaking every 12 h and confirmed by the hemagglutination assay. TCID_50_ was calculated using the Reed-Muench method. Data were analyzed using Prism (v.5.01) software. Statistical significance was set at a *P*-value of <0.05.

### Ethics Statement

These animal studies were performed in strict accordance with the Guidelines for the Care and Use of Animals in Research, which are issued by the Institute of Zoology, Chinese Academy of Sciences (Approval Number IOZ12017).

## Results

### Phylogenetic Analysis and Genetic Characteristics of PPMV-1/BJ-01/CH Isolate

**Figure 1:**
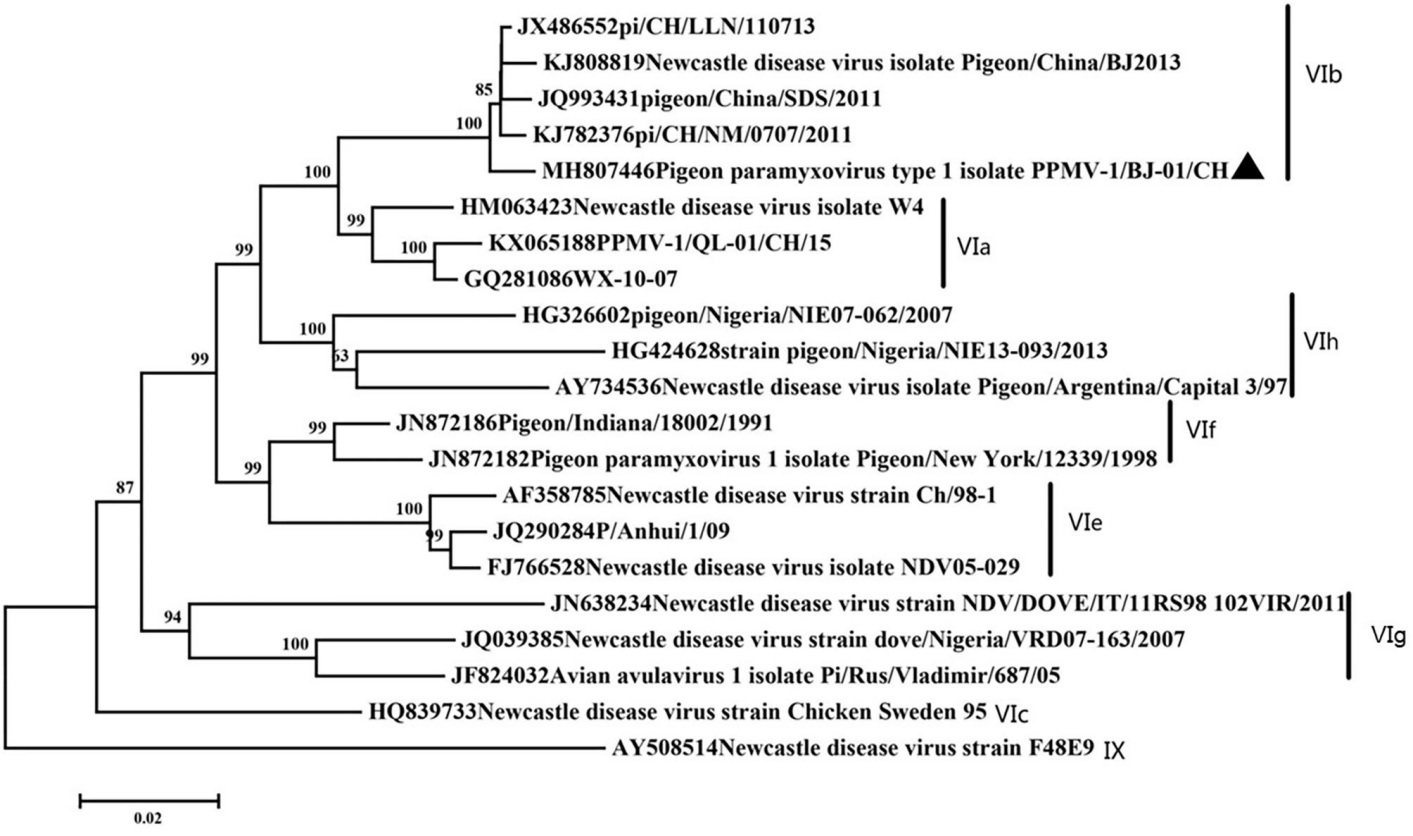
Classification of the virus based on genotype criteria. A maximum likelihood tree of the complete F gene nucleotide sequence. The evolutionary analyses were conducted using the Kimura two-parameter method with 1,000 bootstrap replicates in MEGA7.0. Our isolate was marked with a black triangle.

Phylogenetic analysis showed our isolate belonged to subgenotype VIb (Figure 1). Our isolate possessed an ^11^2R-R-Q-K-RF^117^ segment at the cleavage site of F gene. This is characteristic of virulent NDVs. This motif is commonly found in NDV strains that are highly or moderately virulent in chickens, especially in genotypes VII and IX viruses and some pigeon paramyxovirus strains. Amino acid residues important for receptor recognition of HN is ^174^R ^175^I ^198^D ^236^K ^258^E ^299^Y ^317^Y ^401^E ^416^R ^498^R ^516^R ^526^Y ^547^E (37), which is the same as most PPMV-1 strains.

### Virulence of the PPMV-1/BJ-01/CH Isolate and Sequence Analysis

The MDT and ICPI were 72 h and 0.76, respectively. The results indicated that the PPMV-1 strain was medium according to the criteria (38). Moreover, the minimum lethal dose and 50% egg infection dose of the virus in chick embryo eggs were 10^−3^ and 10^−5.37^, respectively.

### Pathogenicity of the PPMV-1/BJ-01/CH in Different Routes of the Infection

**Figure 2:**
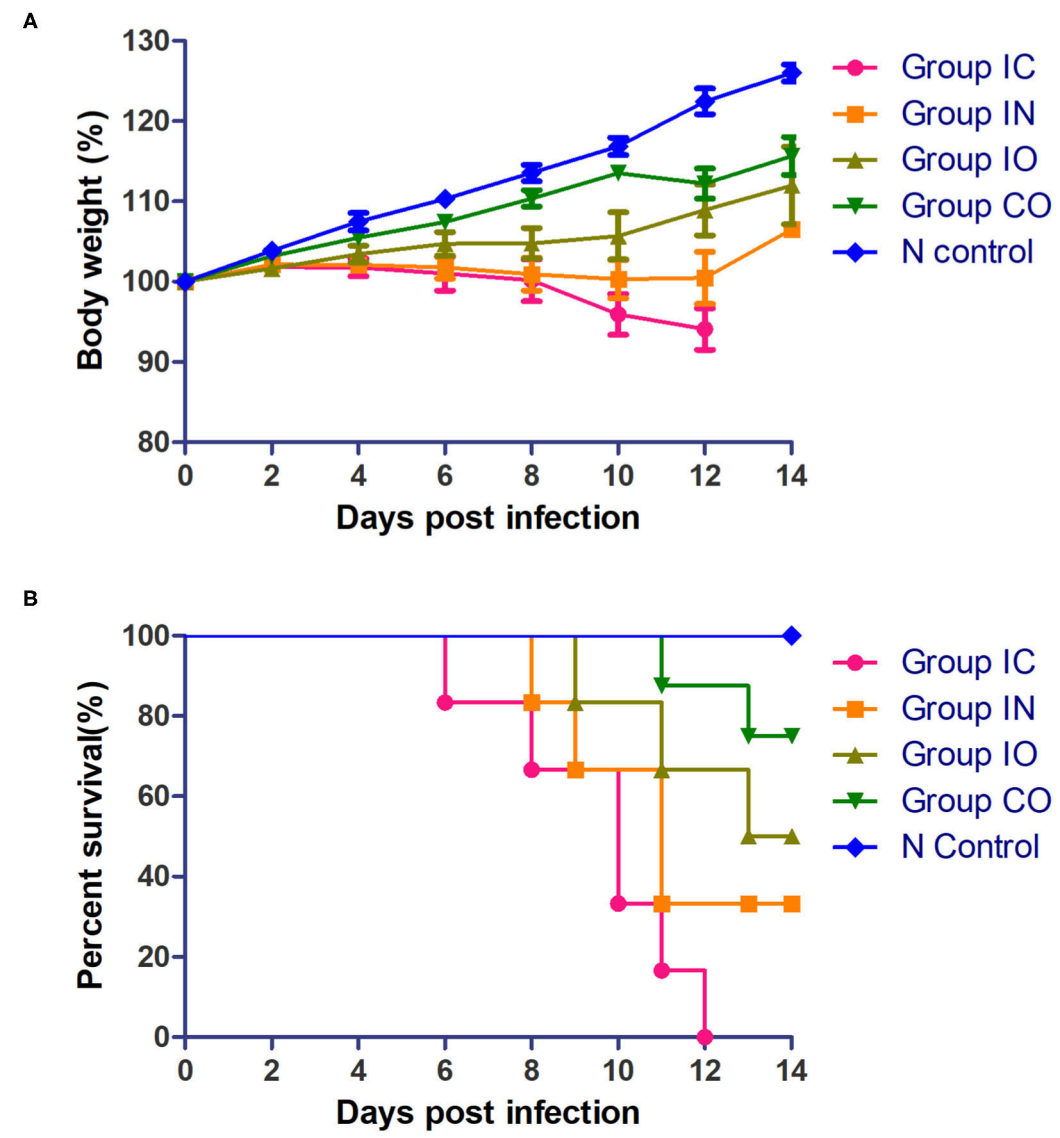
Pathogenicity of the PPMV-1/BJ-01/CH in different routes of infection. **(A)** Body weight change of different groups post-infection. **(B)** Survival curve of different groups post-infection.

Clinical signs were observed in all pigeons from 5 dpi. The pigeons lost their weight sharply once clinical symptoms emerged, then soon died. The body weight change (Figure 2A) and survival curve (Figure 2B) showed the damage of the infected pigeons from group IC were much severer than other groups. A steady increase of body weight was found in negative control group. Post-mortem examination showed the congestion or hemorrhages on the meninx and in the brain. The tracheal mucosa was congested or hemorrhagic. Extensive hemorrhages were observed in the mucosa of the small intestine. The spleens were atrophic, friable, and hemorrhagic. Congestion and hemorrhage were found in the pancreas. Our data showed the most serious tissue lesion was discovered in group IC. Groups IO and CO caused less lesions on dpi 5 (Table 1).

**Table 1:** Percentage of PPMV-1 positive samples as determined by lesions examination.

**Tissues**	**Groups**									
	**IC**		**IO**		**IN**		**CO**		***N*** **control**	
	**D5**	**D14**	**D5**	**D14**	**D5**	**D14**	**D5**	**D14**	**D5**	**D14**
Intestine	67	83	0	33	33	33	0	33	0	0
Lung	67	100	33	50	50	83	13	50	0	0
Brain	33	100	17	67	50	67	0	33	0	0
Liver	50	67	0	33	33	33	0	50	0	0
Heart	17	33	0	0	17	50	17	50	0	0

*The lesions were shown in Supplementary Figure 1*.

**Figure 3:**
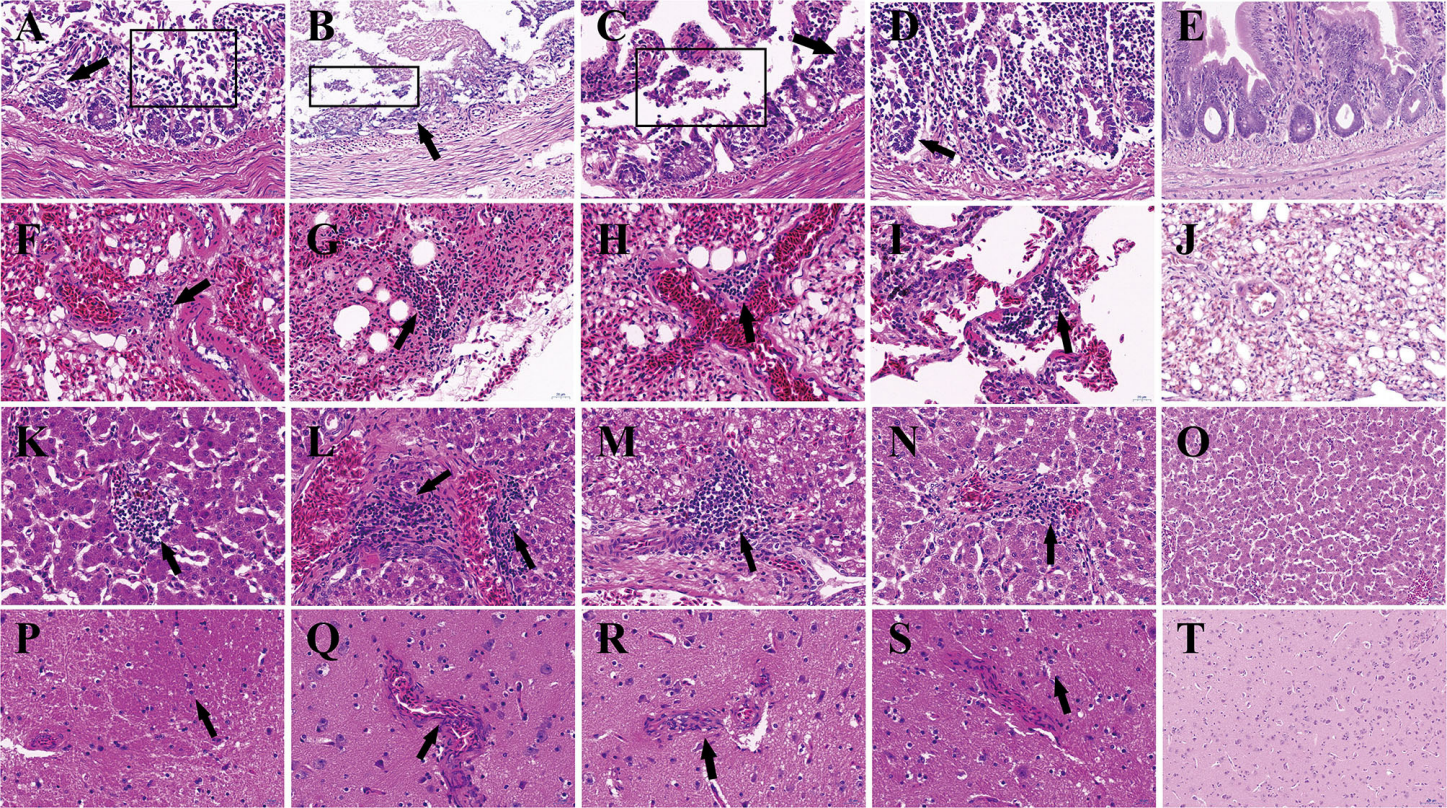
Histopathologic lesions in pigeons of groups. The groups and tissues were marked in the figure. Black arrow indicated the severe histopathologic. **(A,F,K,P)** Histopathologic lesions in group IC. **(B,G,L,Q)** Histopathologic lesions in Group IN. **(C,H,M,R)** Histopathologic lesions in group IO. **(D,I,N,S)** Histopathologic lesions in group CO. **(E,J,O,T)** Histopathologic lesions in group N. **(A–E)** Small intestine. **(F–J)** Lung. **(K–O)** Liver. **(P–T)** Cerebrum. The pictures were magnified ×40.

Histopathological examination showed that the most severe lesions are found in group IC, including the desquamation of small intestine villi, lymphocyte infiltration, and severe epithelial cell loss in the intestine. The alveoli have been destroyed, and a small amount of lymphocyte infiltration and congestion were observed in the lungs. Hepatomegaly and the disappearance of hepatic cord structures were observed in the liver. A small amount of lymphocyte infiltration was also observed in the cerebrum. Milder lesions were observed in other groups (Figure 3).

### Virus Shedding

The results and mortality of each group are compiled in Table 2. Pigeons shed virus from dpi 2 to dpi 14. The virus was detected from oropharyngeal swabs earlier than cloacal swabs in all groups. The earliest start time observed was in groups IC and IN in dpi 2, but the shedding rate was higher in group IN than group IC. The highest shedding rates were observed in group IN from the throat at 2 dpi and cloaca at 7 dpi. Viral shedding ceased at 14 dpi in all groups (Table 2).

**Table 2:** Virus shedding of groups.

**Groups**	**No. of positive samples/No. of pigeons tested Days post-infection**														
	**0**	**1**	**2**	**3**	**4**	**5**	**6**	**7**	**8**	**9**	**10**	**11**	**12**	**13**	**14**
Group IN	0/6^a^	0/6	6/6	6/6	6/6	6/6	6/6	4/6	3/5	2/4	2/4	1/2	1/2	0/2	0/2
	0/6^b^	0/6	0/6	0/6	0/6	0/6	0/6	3/6	2/5	2/4	2/4	0/2	0/2	0/2	0/2
Group IC	0/6	0/6	4/6	6/6	6/6	6/6	5/5	5/5	3/4	3/4	1/2	1/1			
	0/6	0/6	0/6	0/6	1/6	1/6	0/5	0/5	1/4	1/4	1/2	0/1			
Group IO	0/6	0/6	1/6	5/6	4/6	4/6	3/6	3/6	2/6	1/5	0/5	0/4	0/4	0/3	0/3
	0/6	0/6	0/6	0/6	1/6	1/6	2/6	2/6	3/6	3/5	3/5	3/4	3/4	0/3	0/3
Group CO	0/6	0/6	0/6	2/6	5/6	6/6	5/6	2/6	1/6	1/6	1/6	0/6	0/5	0/5	0/5
	0/6	0/6	0/6	0/6	0/6	2/6	2/6	5/6	5/6	4/6	4/6	4/6	3/5	3/5	0/5
*N* control	0/6	0/6	0/6	0/6	0/6	0/6	0/6	0/6	0/6	0/6	0/6	0/6	0/6	0/6	0/6
	0/6	0/6	0/6	0/6	0/6	0/6	0/6	0/6	0/6	0/6	0/6	0/6	0/6	0/6	0/6

a *Positive samples of oropharyngeal swabs*.

b *Positive samples of cloacal swabs*.

### Tissue Tropism of the PPMV-1/BJ-01/CH in Different Routes of Infection

**Figure 4:**
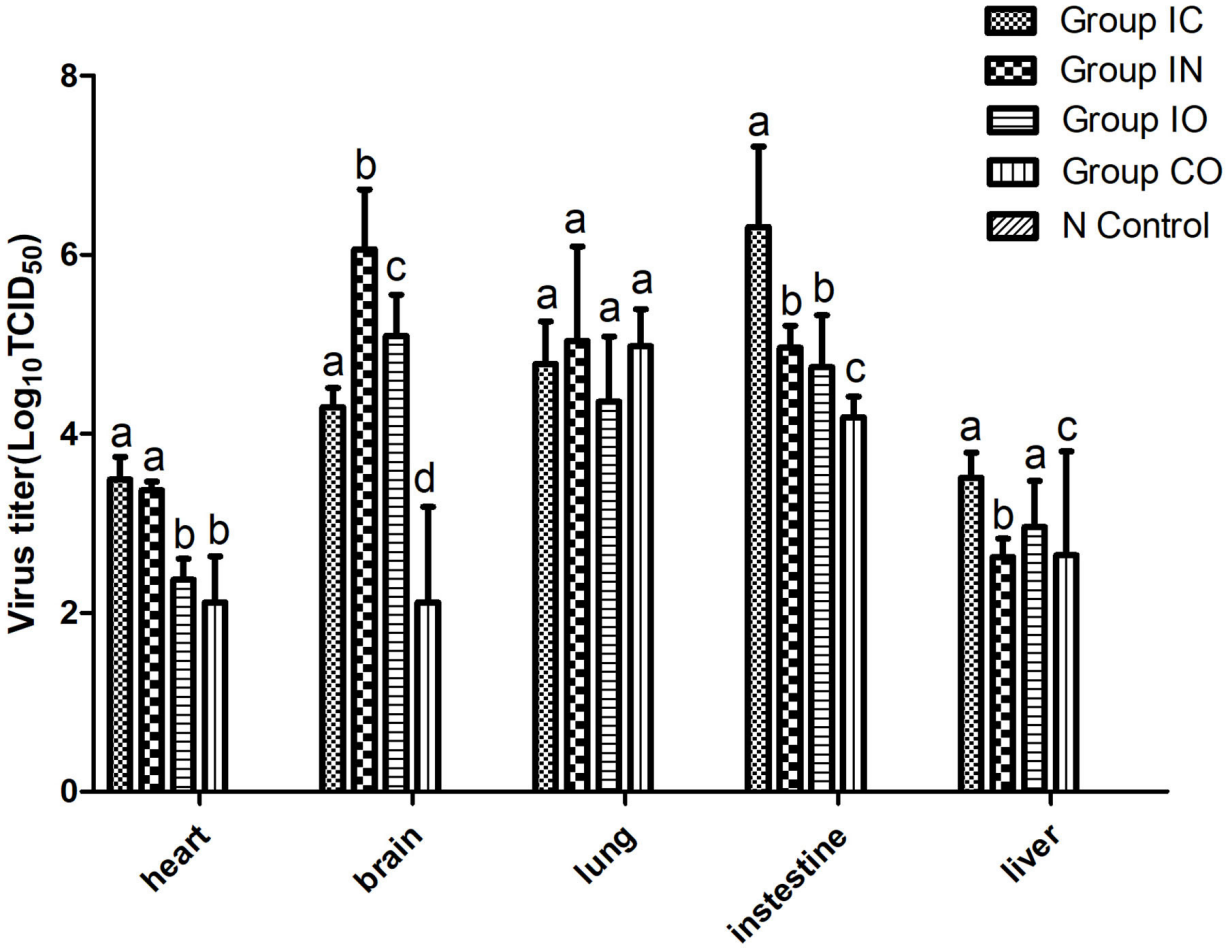
Viral loads in different organs of pigeons post-infection. Virus titer of different tissues from dead pigeons [log_10_ median tissue culture infectious dose/ml (TCID50)] were quantified by serial end-point dilution in 96-well plates using DF-1 cells. The titer was calculated by one-way analysis of variance with standard error bars. Different lowercase letters over the bars denote statistically significant differences (*P* < 0.05) among different tissues after infection calculated by *t*-test. Non-significance (*P* > 0.05) was marked up by the same lowercase letters on different bars. Different lowercase letters marked up on the bars have no relationship among these five organs and represent one organ.

In this study, we found that the virus can effectively replicate in different tissues, no matter which route of infection, and virus titer in the brain, lung, and intestine were higher than in other tissues. The highest titer of the virus was found in the intestine in group IC and brain in group in (Figure 4).

## Discussion

In the present study, a PPMV-1 was isolated from a dead pigeon. In order to better understand the genetic characteristics of the strain, the full-length genome was amplified and sequenced. Phylogenetic analysis showed that the isolate belonged to VIb, class II, which was consistent with most of the genotypes found in China (20, 21, 39–41). Amino acid sequence of F gene 112–117 is a typical virulent motif. But the MDT and ICPI showed medium virulence of our isolate. This contradiction often occurs on PPMV-1. Amino acid residues important for receptor recognition of HN are the same as most PPMV-1. All the characteristics of our strain consisted with current epidemic isolates in China (20, 42, 43).

The pathogenicity of the virus may relate to different inoculation routes. For example, the ducks infected intramuscularly with a virulent NDV strain showed the most severe clinical signs, while ducks infected intranasally and intraocularly sometimes also exhibited clinical signs but seldom died (44). To further understand the target system of the virus obtained in this study, several experiments were conducted to evaluate the pathogenicity *via* different system routes. Cohabitation infection was intended to mimic a natural-acquired infection. Intranasal infection was often used by labs as a substitute for aerosol infection of the virus. Oral infection mimic was used to simulate contaminated food and water. Inoculate intracranially was regularly used to evaluate NDV virulence, so it may be the most effective route of infection.

Viral shedding evaluates the effective replication and transmission of the virus in different groups. The results of our study showed infected pigeons of different groups except group CO which shed virus through the larynx from 2 dpi and through cloaca from 4 dpi. Pigeons shed virus through cloaca from 2 dpi after isolating NDV in previous studies (45, 46). The earliest start time observed was in groups IC and IN, and the shedding rate was higher in group IN than group IC; the highest shedding rates were observed in group IN from the throat at 2 dpi and cloaca at 7 dpi. The result indicated that the respiratory tract is the fastest way for PPMV-1 to spread. The shedding rate in group IC remained high, which may confirm that virus can efficiently enter into the hosts through the nervous system. All infection groups shed virus, and the longest persisted until 14 days. Continuous virus shedding may contribute to circulatory infection. Additionally, we used body weight and survival rate to test the pathogenicity. Death occurred in dpi 6, but previous study of a PPMV-1 was inoculated in pigeons through the intranasal route did not result in mortality up to 31 dpi. (47), it may be caused by the differences in virulence of the isolates used (48). The rate of body weight decline of group IC was higher than in the other groups, followed by groups IN and IO. According to the result of TCID_50_ in DF-1 cells, PPMV-1 was able to cause systemic infection in a relatively short time, and virus titer in groups IC and IN was higher than in other groups, which was broadly consistent with gross lesions at dpi 5, resulting in an effective infection process. Groups IO and CO caused less lesions which might be due to lower viral load in the organs at these days. By comparison of the viral load in different tissues, the virus had a wide range of tissue distribution, especially in the lung, brain, and intestine. It can be inferred that the virus could replicate well in these three organs during early infection. At 14 dpi, the gross lesions were more severe in these groups, but the rate of virus shedding was reduced which may be due to higher mortality rates. The results indicated that the virus can effectively infect pigeon *via* different routes, and the most pathogenic was infection through the nervous system and respiratory system, but infection through the nervous showed stronger pathogenicity. Previous studies described neurological lesions in NDV-infected birds, and virulent virus was capable of replication in the brain, but not the avirulent virus (49–51), thus successful replication in the nervous system determines its pathogenicity.

Overall, pigeons play an important role in the epidemiology of PPMV-1. The routes of inoculation greatly influenced pathogenicity of the pathogenic strain isolated in the present study. Considering the growing number of PPMV-1 cases in recent years, it is necessary to develop effective vaccines or other prevention and control methods.

## Data Availability Statement

Complete genome of our strain was amplified (primers were showed in Supplementary Table 1), sequenced and submitted to GenBank (GenBank ID: MH807446).

## Ethics Statement

The animal study was reviewed and approved by Institute of Zoology, Chinese Academy of Sciences (Approval Number IOZ12017).

## Author Contributions

HH and HC designed the experiments. HC and SF conducted the experiments and analyzed the data. YW and FL provided the animals. BW performed the RNA extraction and PCR. HC wrote the manuscript. SF, QS, and JD revised the manuscript. All authors read and approved the final version of the manuscript.

## Conflict of Interest

The authors declare that the research was conducted in the absence of any commercial or financial relationships that could be construed as a potential conflict of interest.
