# Supplementary material for: Isolation and Pathogenic Characterization of Pigeon Paramyxovirus Type 1 via Different Inoculation Routes in Pigeons
Source: Front Vet Sci. 2021 Feb 17;7:569901. doi: 10.3389/fvets.2020.569901 (PMC7925627; doi:10.3389/fvets.2020.569901)
Supplement: Supplementary file 1 [file Table_1.docx]

**Table S1:** Genome primer sequence of PPMV-1.

| Primer name | Primer sequence | Position | Length (bp) | Annealing temperature |
| --- | --- | --- | --- | --- |
| 1F | GCTCAGACTCGCCCTAAT | 161-1532 | 1371 | 55°C |
| 1R | GATGAATGGTGCTCTGTG |  |  |  |
| 2F | CTTACTGGGCTCAGCGACAA | 1367-2592 | 1225 | 55°C |
| 2R | ACGCCTCCATCATAGACA |  |  |  |
| 3F | GAGCAGAGCCAAGACAGT | 2502-3546 | 1044 | 55°C |
| 3R | TGTCTGGGATTGTCGTTG |  |  |  |
| 4F | TTGGCGGCATTCTGGTTAGC | 3104-4998 | 1894 | 53°C |
| 4R | CTCCCGCCTCATTAGTGTCA |  |  |  |
| 5F | CAAGGTTGCCTGTCACGAT | 4666-5933 | 1267 | 54°C |
| 5R | TGCGGGGATTGTGGTAACA |  |  |  |
| 6F | GAACCAGACACGGTCATCA | 5681-7359 | 1678 | 54°C |
| 6R | GCACTCAATACGCCATACA |  |  |  |
| 7F | GCACAGCTTGGCGAACAGG | 7060-8782 | 1722 | 53°C |
| 7R | GCAACAGGGAGGGTATTCT |  |  |  |
| 8F | TGATAGCATGGGCGAATAGC | 8507-10370 | 1863 | 52°C |
| 8R | CCTGATGAGTGCGACTTTGA |  |  |  |
| 9F | ACTTGAATCTGCCACTCC | 8560-9668 | 1108 | 51°C |
| 9R | GCTGCCCAATGACCTTCC |  |  |  |
| 10F | TAAGGGAACAATCATCAA | 9574-10648 | 1074 | 47°C |
| 10R | TACCTGATTGTCACCTTG |  |  |  |
| 11F | CTATCAAGAGTCCCAAAT | 10484-11707 | 1223 | 48°C |
| 11R | AACTAATGGGTGGTTGGA |  |  |  |
| 12F | TCAAGAGGCTGACACGAA | 11610-12778 | 1168 | 49°C |
| 12R | GAATAGGCGAACCACATC |  |  |  |
| 13F | CGTGGTTTCCTATGATGA | 12667-13739 | 1072 | 50°C |
| 13R | TGATGGCTCCACTTCCTT |  |  |  |
| 14F | ACGGTATTTGTTCAGAGG | 13606-14743 | 1137 | 48°C |
| 14R | GGCTCTGAGACCCAATAT |  |  |  |
| 15F | ATGGAGGCTGAAGGTGAC | 14606-15182 | 576 | 50°C |
| 15R | GATTTGGTGAACGATGGG |  |  |  |
