# Supplementary figures and images for: Isolation and Pathogenic Characterization of Pigeon Paramyxovirus Type 1 via Different Inoculation Routes in Pigeons
Source: Front Vet Sci. 2021 Feb 17;7:569901. doi: 10.3389/fvets.2020.569901 (PMC7925627; doi:10.3389/fvets.2020.569901)

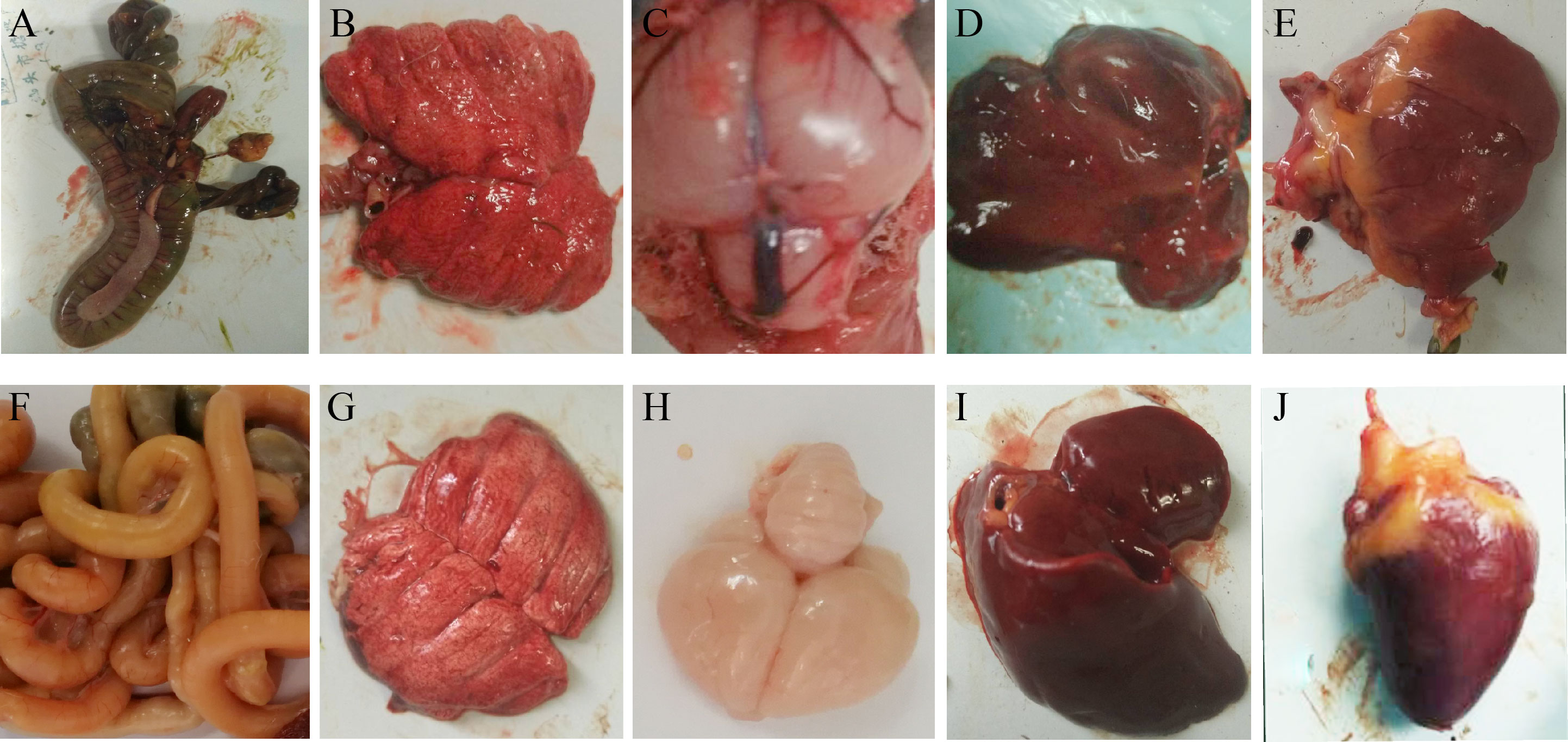

Supplement: Supplementary file 2 [file Image_1.jpg]
